# Exosomal miR-21 regulates the TETs/PTENp1/PTEN pathway to promote hepatocellular carcinoma growth

**DOI:** 10.1186/s12943-019-1075-2

**Published:** 2019-10-27

**Authors:** Liang-qi Cao, Xue-wei Yang, Yu-bin Chen, Da-wei Zhang, Xiao-Feng Jiang, Ping Xue

**Affiliations:** Department of Hepatobiliary Surgery, the Second Affiliated Hospital of Guangzhou Medical University, 250# Changgang East Road, Haizhu District, Guangzhou, 510260 People’s Republic of China

**Keywords:** Hepatocellular carcinoma, Exosome, miR-21, TET, PTEN, PTENp1

## Abstract

**Background:**

As an important means of communication, exosomes play an important role in the development of hepatocellular carcinoma (HCC).

**Methods:**

Bioinformatics analysis, dual-luciferase reporter assays, methylation-specific quantitative PCR, and ChIP-PCR analysis were used to gain insight into the underlying mechanism of miR-21 in HCC.

**Results:**

The detection of miRNAs in exosomes of HCC showed that miR-21 expression in exosomes was positively correlated with the expression level of miR-21 in cells and negatively correlated with the expression of its target genes PTEN, PTENp1 and TETs. HCC cell-derived exosomes could increase miR-21 and p-Akt expression in HCC cells and downregulate the expression of PTEN, PTENp1 and TETs. MiR-21 inhibitors or PTENp1 overexpression vectors could weaken the effect of the abovementioned exosomes and simultaneously weaken their role in promoting cell proliferation and migration and inhibiting apoptosis. Further studies showed that miR-21 not only directly regulated the expression of PTEN, PTENp1 and TETs but also increased the methylation level of the PTENp1 promoter by regulating the expression of TETs, thereby inhibiting the expression of PTENp1 and further downregulating the expression of PTEN.

**Conclusions:**

Exosomal miR-21 can regulate the expression of the tumor suppressor genes PTEN and PTENp1 in various ways and affect the growth of HCC cells.

## Introduction

Hepatocellular carcinoma (HCC) is one of the most common malignant tumors in the world and ranks fifth in incidence and third in mortality [1]. Therefore, it is necessary to study the molecular mechanism of the occurrence and development of HCC and the signaling pathways that regulate tumor invasion and metastasis.

Exosomes are membrane vesicle-like bodies secreted by cells into the extracellular space and are important carriers of material and mediators of information exchange between cells [2, 3]. Studies have shown that exosomal miRNAs, long noncoding RNAs (lncRNAs), and proteins can mediate the transfer of biological information between the tumor and tumor microenvironment and participate in the biological process of HCC in many ways [2–4]. Multiple studies have shown that miR-21 is elevated in both HCC- and HCC-derived exosomes [5–7]. An increasing number of experiments have shown that miR-21 is the only miRNA that is highly expressed in almost all solid cancers and is also elevated in various tumor-derived exosomes [7, 8]. MiR-21 plays an anti-apoptotic, pro-survival role in tumor cells and plays an important role in tumor biology, diagnosis and prognosis [8]. Therefore, exosomal miR-21 may have a wide range of regulatory roles in the development of tumors.

Phosphatase and tensin homolog (PTEN) is an important target gene of miR-21, which inhibits tumor cell apoptosis and increases tumor cell growth, metastasis and invasion by downregulating the expression of PTEN [9]. In many tumor tissues, miR-21 is negatively correlated with PTEN expression [10]. PTEN is a tumor suppressor gene with bispecific phosphatase activity, and its expression is generally decreased in liver cancer and other tumors [11]. The expression of PTEN is also regulated by its pseudogene PTENp1 (PTEN pseudogene 1). It was found that lncRNA PTENp1 could compete with the tumor suppressor gene PTEN for binding to multiple miRNAs and block the posttranscriptional inhibitory effect of these miRNAs on PTEN mRNA, thus ensuring the normal expression of PTEN [12]. Yu et al. [12] found that the expression of PTENp1 was generally low or undetectable in clinical samples of primary clear cell renal cell carcinoma due to methylation and was positively correlated with the expression of the tumor suppressor gene PTEN.

Although the expression of PTEN and PTENp1 is generally downregulated in tumor cells, it has been found that the promoter that is methylated in tumor cells is mainly that of PTENp1 not PTEN [13]. Hypermethylation of the promoter region is the most common cause of tumor suppressor gene inactivation in malignant tumors. There is a dynamic balance between promoter methylation catalyzed by DNA methyltransferases (DNMTs) and active demethylation catalyzed by Tet methylcytosine dioxygenases (TETs) [14]. An increasing number of studies have found that the expression of TETs is downregulated in breast cancer, liver cancer, lung cancer, pancreatic cancer and prostate cancer [15]. However, whether the downregulation of TETs affects the methylation of the PTENp1 promoter has not been studied. Bioinformatic analysis showed that miR-21 had binding sites on PTEN, PTENp1 and TET family proteins (TET1, TET2 and TET3). Therefore, we will conduct an in-depth study to determine if, in addition to directly regulating the expression of PTEN and PTENp1, miR-21 can also indirectly affect the expression of PTEN and PTENp1 by regulating the expression of TET family genes.

## Materials and methods

### Cell culture

Human hepatocellular cancer cell lines (SNU-449, HepG2 and Hep3B cells) were obtained from American Type Culture Collection (ATCC; Manassas, VA, USA), and the Huh7 liver cancer cell line was purchased from the Cell Bank of the Chinese Academy of Sciences. All cell lines were cultured in Dulbecco’s modified Eagle’s medium (DMEM; Invitrogen, Carlsbad, CA) with 10% fetal bovine serum (Invitrogen). Noncancerous primary human hepatocytes were purchased from Lonza Inc. (Walkersville, MD) and were cultured in collagen I-coated plates (BD Biosciences, Bedford, MA) with hepatocyte basal medium supplemented with HCM SingleQuots growth factors (Lonza Inc.). The cells were maintained at 37 °C and 5% CO_2_.

### Exosome purification

Five micrometers GW4869 (Sigma-Aldrich, MO, USA) was added to the cells for culture if necessary, and exosomes were isolated from the cell culture supernatant by differential centrifugation. In brief, 2.5 × 10^6^ HCC cells were seeded in 150 mm dishes and allowed to recover for 24 h. Then, the cells were washed twice with prewarmed phosphate-buffered saline (PBS), and the culture medium was replaced with exosome-free medium supplemented with 10% exosome-depleted FBS. After 2 days of culture, the conditioned medium from cells reaching ~ 90% confluence was harvested and subjected to serial centrifugations for 10 min at 500 g and 30 min at 16500 g, followed by filtration through a 0.22 μm pore filter (Millipore). The filtrated medium was ultracentrifuged at 110000 g for 70 min to harvest exosomes. The exosome pellet was washed with PBS and then collected by ultracentrifugation at 110000 g for 70 min on a 40% w/v sucrose cushion. The floating exosomes were collected and pelleted again by ultracentrifugation at 110000 g for 70 min. High-resolution transmission electron microscopy (HR-TEM) of the exosomes was carried out with a JEOL 2010 microscope (Akishima, Japan) at 200 kV.

### Fluorescence microscopy analysis of exosome internalization

SNU-449-derived exosomes were labeled with CM-Dil (Sigma-Aldrich, St. Louis, MO, USA) as follows: 2 μl CM-Dil was added to 100 μg exosomes in a total of 1 mL diluent and incubated for 15 min at room temperature, and the mixture was added to 18 mL PBS and centrifuged at 120000 g for 2 h at 4 °C. The supernatant was removed, and the pellet was resuspended in 20 mL PBS and centrifuged at 120000 g for 2 h at 4 °C. The pellet containing CM-Dil-labeled exosomes was resuspended in 200 μL PBS and then added to the cells. After incubation for 24 h, the cells were washed twice with PBS and fixed in polyformaldehyde for 10 min, and internalization of the exosomes was analyzed using fluorescence microscopy.

### Transfection and quantitative RT-PCR

Cells were seeded in 6-well plates at 1 × 10^5^ cells/mL/well 1 day before the transfection. On the following day, transfection was performed when the cells had reached approximately 70% confluence. miR-21 mimics and inhibitors and PTENp1, TET1, TET2 and TET3 siRNAs (Table 1) were purchased from GenePharma (Shanghai, China). PTENp1, TET1, TET2 and TET3 overexpression vectors and lentivirus encoding human miR-21, miR-21 inhibitors, PTEN shRNA, TET1, TET2 or TET3 were purchased from GeneCopoeia (Guangzhou, China). The final concentrations of plasmid and miRNA/siRNA were 2.0 μg/mL and 50 nM, respectively. Transfections were conducted with Lipofectamine 2000 (Invitrogen) according to the manufacturer’s instructions. The transfection medium was replaced 4–6 h after transfection. Five micrometers MK-2206 (an Akt inhibitor; Selleck, TX, USA) was added to the cells for culture if necessary. Total RNA was extracted using a TRIzol plus RNA purification kit (Thermo Fisher Scientific, China) and was reverse transcribed to cDNA using SuperScript II reverse transcriptase (Thermo Fisher Scientific). The cDNA samples were used for quantitative RT-PCR analysis in triplicate to determine the expression levels of miR-21, PTEN, PTENp1, TET1, TET2 and TET3 using a QuantiFast SYBR Green PCR Kit (Qiagen, Valencia, CA) and an ABI 7500 real-time PCR instrument.

**Table 1:** Sequences of primers and siRNAs

Name	sense (5′-3′)	antisense (5′-3′)
Primer set for real-time PCR		
GAPDH	AGGTCGGTGTGAACGGATTTG	TGTAGACCATGTAGTTGAGGTCA
PTEN	CTTACAGTTGGGCCCTGTACCATCC	TTTGATGCTGCCGGTAAACTCCACT
PTENp1	GGATCATTACCTCACACCATACC	TCTAAGAAACAACTAAGCCAAAGTC
TET1	TCTGTTGTTGTGCCTCTGGA	GCCTTTAAAACTTTGGGCTTC
TET2	GAGACGCTGAGGAAATACGG	TGGTGCCATAAGAGTGGACA
TET3	CCCACAAGGACCAGCATAAC	CCATCTTGTACAGGGGGAGA
MS QPCR primers		
unmethylated PTENp1	TAGTAGTGAGAATATTTGGATATAGGGC	AATTTACTACACCGATTAACTCGTC
methylated PTENp1	GAGAATATTTGGATATAGGGTGG	AATTTACTACACCAATTAACTCATC
ChIP-PCR primers	ATTACGAACCTAGAAGATGCTCTC	CTCACAGCGGCTCAACATTC
GlucMS-qPCR primers	ACGAACCTAGAAGATGCTCTCCTC	AGCCCGGCCTCGCCTCAC
miR-21 inhibitor	UAGCUUAUCAGACUGAUGUUGA	AUCGAAUAGUCUGACUACAACU
siRNAs		
PTENp1	ATCAGAGATCATATAGGAATA	
TET1	CCAGTCTTAATAAGGTTAT	
TET2	CAAGACCAATGTCAGAA	
TET3	GATGAAGGTCCATATTA	
Negative control	AACAGTCGCGTTTGCGACTGG	

### Western blot analysis

The cells were collected, washed twice with PBS, and lysed in RIPA buffer containing protease inhibitors. After determining the protein concentration with a BCA Protein Assay Reagent Kit (Pierce), equal amounts of protein were separated on 8% SDS-PAGE, electrically transferred to PVDF membrane, and blocked with 5% skim milk. The membranes were incubated with anti-CD63 (1:500; Abcam, China), anti-TSG101 (1:800; Abcam), anti-CD81 (1:1000; Abcam), anti-CD9 (1:1000; Cell Signaling Technology, China), anti-ENO1 (1:800; Cell Signaling Technology), anti-KRT19 (1:800; Cell Signaling Technology), anti-ANXA1 (1:800; Cell Signaling Technology), anti-PTEN (1:800; Cell Signaling Technology, China), anti-TET1 (1:500; Novus Biologicals, China), anti-TET2 (1:500; Cell Signaling Technology), anti-TET3 (1:500; Novus Biologicals), anti-Akt (1:1000; Cell Signaling Technology), anti-p-Akt (1:1000; Cell Signaling Technology) or anti-beta-actin (1:1000; Cell Signaling Technology) primary antibody overnight at 4 °C. After washing, the membranes were then incubated with horseradish peroxidase (HRP)-conjugated anti-rabbit secondary antibody (Cell Signaling Technology) at room temperature for 1 h. Finally, the membranes were incubated with West Femto chemiluminescence substrate (Pierce), and images were visualized and recorded.

### Dual-luciferase assay

The 3′ untranslated region (UTR) sequences of TET1, TET2 or TET3 were cloned into the dual-luciferase reporter vector psiCHECK-2 (Promega, China). Each of the above plasmids and miR-21 mimics were cotransfected into cells, and changes in luciferase expression were analyzed by using the Dual Glo Luciferase Assay System (Promega) following the manufacturer’s instructions.

### Methylation-specific quantitative PCR

Methylation-specific real-time qPCR primers (Table 1) for CpG-rich regions were designed using Methyl Primer Express v1.0 software (Applied Biosystems, CA, USA). Quantification of DNA methylation status was determined using the EpiTect Methyl qPCR assay (SABiosciences, Frederick, MD) by following the manufacturer’s protocol. Briefly, gDNA was digested with a combination of methylation-sensitive, methylation-dependent, and both methylation-sensitive and methylation-dependent enzymes or without enzyme added (mock) at 37 °C for 16 h. After enzyme inactivation at 65 °C for 20 min, real-time qPCR was carried out according to the EpiTect protocol. All reactions were performed in triplicate. Relative fractions of methylated and unmethylated DNA were measured by comparing the amount in each digest with that of the mock digest using the ΔCt method.

### Chromatin immunoprecipitation (ChIP)-PCR analysis

ChIP was performed in native conditions. Briefly, cells at a concentration of 2 million cells/mL were treated with 1% formaldehyde in medium for 10 min at room temperature. After two washes with ice-cold PBS containing protease inhibitors, the cells were pelleted by centrifugation and resuspended in SDS lysis buffer. After incubation for 15 min at 4 °C, the lysates were sonicated 12 times (30 s each). After centrifugation, the supernatant was diluted in ChIP dilution buffer and incubated overnight at 4 °C with anti-TET1, anti-TET2 or anti-TET3 antibody and protein G beads. Samples were washed two times in lysis buffer, four times in 1 M lysis buffer (50 mM Tris, pH 7.4, 1 M NaCl, 1 mM EDTA, 0.1% SDS, 1% NP-40, and 0.5% sodium deoxycholate), and the beads were then resuspended in lysis buffer and treated with proteinase K at 45 °C for 45 min. Coprecipitated DNAs were purified using a QIAquick DNA purification spin column (Qiagen, Germantown, MD, USA) and eluted in 50 μL nuclease-free water. The immunoprecipitated DNA was quantified using PCR, and all values were normalized to the input.

### Quantification of 5hmC levels in gDNA by methylation-sensitive qPCR

gDNA was incubated with T4 Phage β-glucosyltransferase (New England Biolabs, Ipswich, MA) by following the manufacturer’s protocol. First, 100 ng of glucosylated gDNA was digested with Hpa II, Msp I, or without enzyme (mock) at 37 °C overnight and then incubated for 20 min at 80 °C for enzyme deactivation. The Hpa II- or Msp I-resistant DNA fraction was quantified by qPCR and normalized to the mock control. Msp I-resistant DNA represents the 5hmC DNA fraction, whereas the fraction of 5mC DNA was calculated by subtracting the 5hmC fraction from the Hpa II-resistant DNA.

### Cell proliferation assay

A BrdU colorimetric immunoassay kit (Cell Proliferation ELISA, Roche Diagnostics, Germany) was used for quantification of cell proliferation according to the protocol provided by the manufacturer. Cell proliferation was expressed as the mean percentage of the control values (set at 100%).

### Flow cytometric analysis of apoptosis

Cell apoptosis was analyzed using an Annexin V-fluorescein isothiocyanate (FITC) Apoptosis Detection Kit (KGI, China) in accordance with the manufacturer’s instructions. After a 48-h transfection the cells were harvested and then resuspended in 500 μL of binding buffer with 5 μL of Annexin V-FITC solution. Subsequently, 5 μL of propidium iodide (PI) was added to the cell suspension, and the cells were stained at room temperature for 15 min in the dark. After double staining with Annexin V-FITC and PI, flow cytometry (FACScan; BD Biosciences, CA, USA) was used to analyze the cells.

### Determination of cell invasion by Transwell assay

The Transwell chambers were coated with Matrigel (BD). The cells were collected on the second day after transfection and adjusted to a density of 1 × 10^6^ cells/mL. The cells (200 μL) were added onto the upper chamber of the Transwell insert, and 600 μL of complete medium was added to the lower chamber. After 48 h of culture at 37 °C and 5% CO_2_, the cells were fixed in 4% paraformaldehyde for 15 min, washed once with PBS, stained with crystal violet for 10 min, washed with PBS once, and finally imaged and counted.

### Determination of tumorigenicity in nude mice

Animal care and use followed the ethical guidelines of the Chinese Council on Animal Care and were reviewed and approved by the Institutional Animal Care and Use Committee. A total of 5 × 10^6^ SNU-449 cells and/or exosomes were suspended in 100 μL serum-free DMEM and Matrigel (BD Biosciences, Franklin Lakes, NJ, USA) at a (1:1 ratio) and then injected at a single subcutaneous site of the left armpit into each 4-week-old nude mouse (BALB/c, Experimental Animal Center of Sun Yat-Sen University). All mice were examined regularly for development of tumors until the tumor diameter was more than 1 cm or the experimental period reached 60 days. The mice were sacrificed by cervical dislocation, and the tumors were removed.

### Statistical analysis

The experiments were carried out at least in triplicate, and the results are expressed as the mean ± SD. Statistical analysis was performed using the SPSS 17.0 statistical software (Chicago, IL, USA). Differences between two groups were analyzed by two-tailed Student’s *t*-test, and differences between three or more groups were analyzed by one-way ANOVA with multiple comparisons. Differences at *P* < 0.01 (*) levels were considered statistically significant.

## Results

### HCC cells secrete exosomes with high levels of miR-21, and miR-21 has an inverse relationship with PTENp1 and PTEN expression

**Fig. 1:**
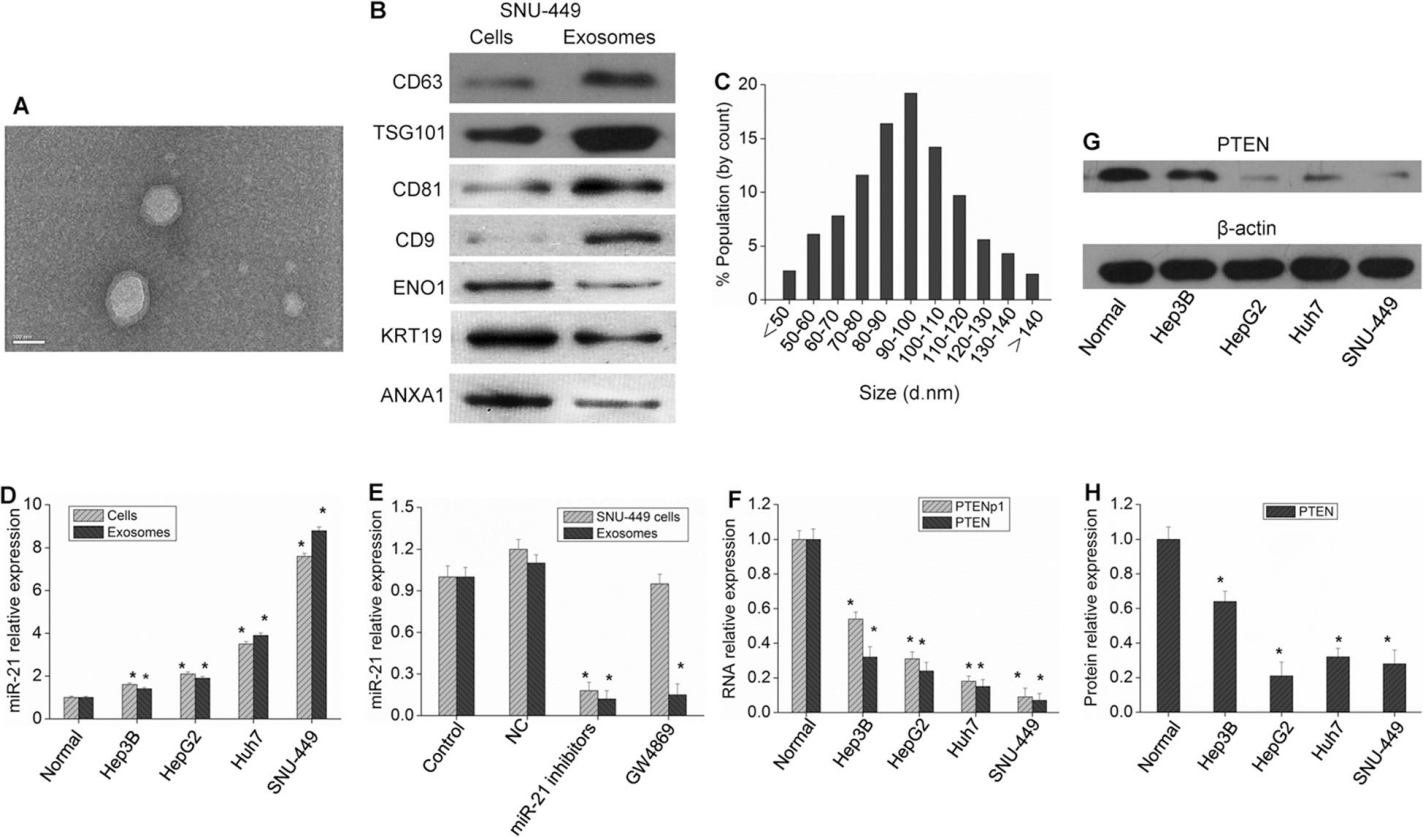
Characterization of exosomes and the expression of miR-21, PTENp1 and PTEN. **a** High-resolution transmission electron microscopic image of SNU-449 cell exosomes (scale bar = 100 nm). **b** Western blot analysis showing the expression of exosomal biomarkers in HCC cells and exosomes. **c** The size distribution of isolated exosomes. **d** MiR-21 expression in noncancerous primary human hepatocytes (normal), HCC cells and exosomes was measured by real-time qPCR analysis. **e** After transfection of miR-21 inhibitors or addition of GW4869, miR-21 expression in SNU-449 cells and exosomes was measured by real-time qPCR analysis. **f** PTEN and PTENp1 expression in hepatocytes and HCC cells was measured by real-time qPCR analysis. **g** and **h** PTEN expression was measured by western blot analysis. The values expressed are the mean ± SD from three samples (^*^P < 0.01, vs. normal or control)

The secretion of exosomes in normal hepatocytes and HCC cells was detected, and the results showed that both types of cells could secrete exosomes that expressed exosome-specific markers (Fig. 1a, b), and their diameters were mainly between 70 and 120 nm (Fig. 1c). The expression of miR-21 was generally upregulated in HCC cells, but its expression in HCC cells from different sources was significantly different, and miR-21 expression was also significantly upregulated in exosomes derived from HCC cells with high expression of miR-21 (Fig. 1d). But miR-21 inhibitors and GW4869 (an exosome release inhibitor) significantly inhibited the expression of miR-21 in exosomes (Fig. 1e). It has been demonstrated that PTEN and PTENp1 are both target genes of miR-21. The expression of PTEN and PTENp1 in HCC cells was inversely related to that of miR-21 and was lower in HCC cells with higher expression of miR-21 (Fig. 1f-h).

### HCC cells can uptake exosomes with high expression of miR-21 which affects their growth

**Fig. 2:**
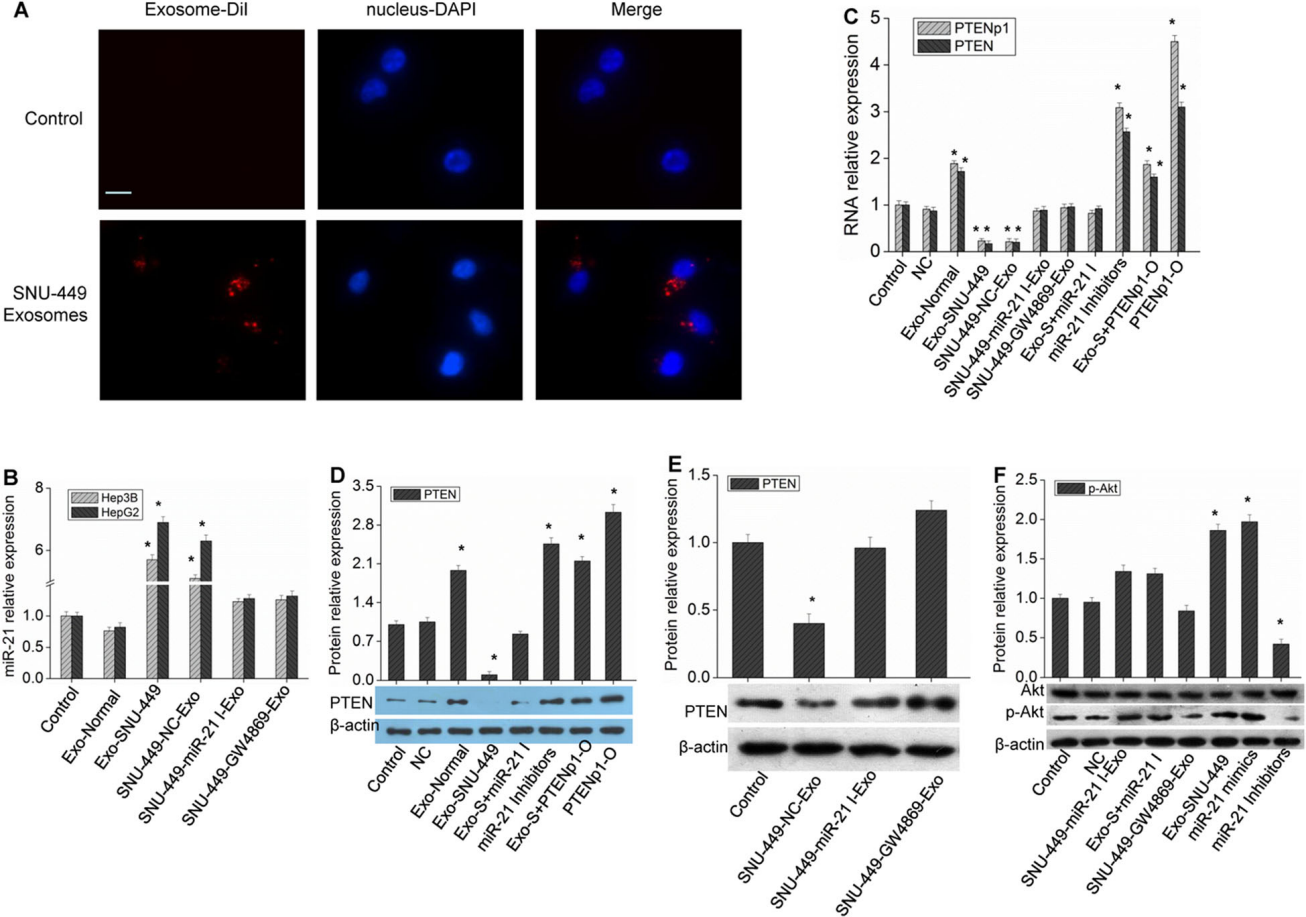
Exosomal miR-21 regulates the expression of PTEN and PTENp1 in HCC cells. **a** SNU-449 cell exosomes stained with Dil were added to culture medium of Hep3B cells, and the cells were observed and imaged under a confocal microscope. Scale bar = 25 μm. **b** After adding exosomes obtained from hepatocytes (Exo-normal), SNU-449 cells (Exo-SNU-449), SNU-449 cells transfected with negative control (NC) miRNA (SNU-449-NC-Exo), SNU-449 cells transfected with miR-21 inhibitors (SNU-449-miR-21 I-Exo) or SNU-449 cells cultured with GW4869 (SNU-449-GW4869-Exo) to Hep3B or HepG2 cells for 24 h, miR-21 expression was measured by real-time qPCR analysis. **c** Hep3B cells were transfected with miR-21 inhibitors (miR-21 I) or a PTENp1 overexpression vector (PTENp1-O), then Exo-SNU-449 (Exo-S) were added to the cells, and PTEN and PTENp1 expression was measured by real-time qPCR analysis. (DF) After different treatments, PTEN (**d** and **e**), Akt and p-Akt (**f**) expression was measured by western blot analysis. Each bar represents the mean ± SD determined from three samples (^*^P < 0.01, vs. control)

**Fig. 3:**
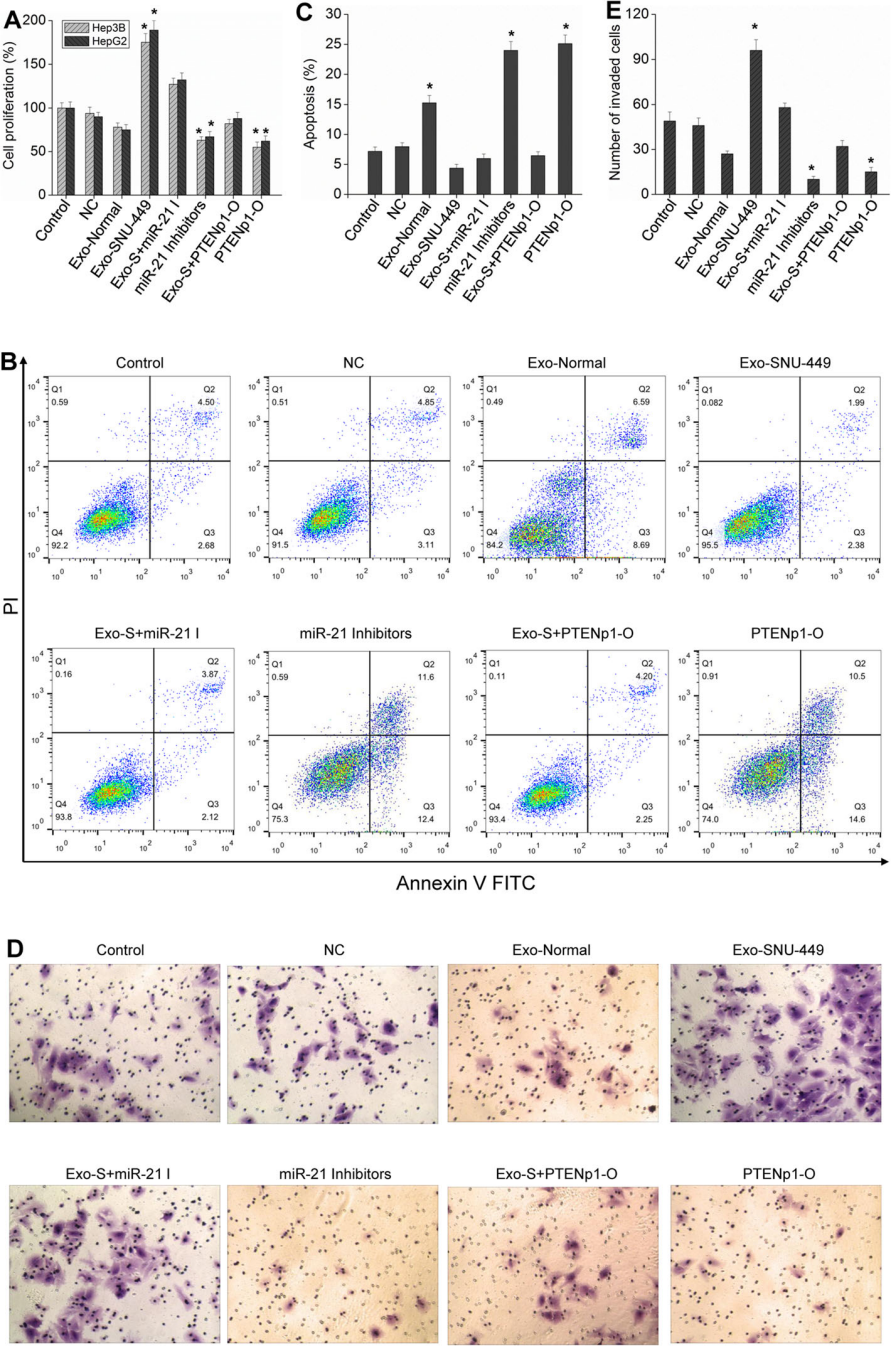
Exosomal miR-21 regulates the expression of PTEN and PTENp1 to affect HCC cell growth. **a** After different treatments, cell proliferation was examined by BrdU assay. **b** and **c** Cell apoptosis was detected by flow cytometric analysis. **d** and **e** Invasion of cells was evaluated by Transwell Matrigel invasion assay (200 × magnification). Each bar represents the mean ± SD determined from three samples (^*^P < 0.01, vs. control)

The exosomes secreted from SNU-449 cells were isolated and purified and added to Hep3B or HepG2 cells for culture. The results showed that the cells could absorb the exosomes (Fig. 2a). Then, Hep3B or HepG2 cells were collected to detect the changes in miR-21 expression in the cells. Compared with that of the control group, the expression of miR-21 in HCC cells cultured with exosomes was significantly increased (Fig. 2b), indicating that HCC cells could uptake exosomes. However, after transfection of miR-21 inhibitors or addition of GW4869, the collected exosomes had no such effect (Fig. 2b). The effect of exosomes on PTENp1 and PTEN expression was also analyzed. The results showed that PTENp1 and PTEN expression decreased significantly after the cells were incubated with exosomes with high expression of miR-21, and the effect of these exosomes was blocked by adding miR-21 inhibitors or a PTENp1 overexpression vector (Fig. 2d-e). PI3K/Akt is an important signaling pathway downstream of PTEN, and the results further found that exosomes from SNU-449 cells increased the phosphorylation of Akt significantly, but miR-21 inhibitors or GW4869 could block this effect (Fig. 2f). Experiments measuring the proliferation and apoptosis of HCC cells showed that exosomes rich in miR-21 could significantly promote the proliferation of HCC cells, inhibit their apoptosis, and promote cell invasion (Fig. 3). Similarly, the effect of the abovementioned exosomes on HCC cell growth could be reversed by adding miR-21 inhibitors, a PTENp1 overexpression vector or an Akt inhibitor MK-2206 (Fig. 3, Additional file 1: Figure S1). Therefore, the exogenous exosomes taken up by HCC cells affect cell growth by releasing highly expressed miR-21, thereby regulating the expression of PTENp1 and PTEN. In addition, after PTEN was knocked down by infecting cells with lentiviruses containing PTEN shRNA, miR-21 still had the effect of promoting growth (Additional file 1: Figure S2), indicating that miR-21 could also play a role by targeting other cancer suppressor genes.

### PTENp1 expression is also influenced by the demethylation mechanism regulated by TETs

**Fig. 4:**
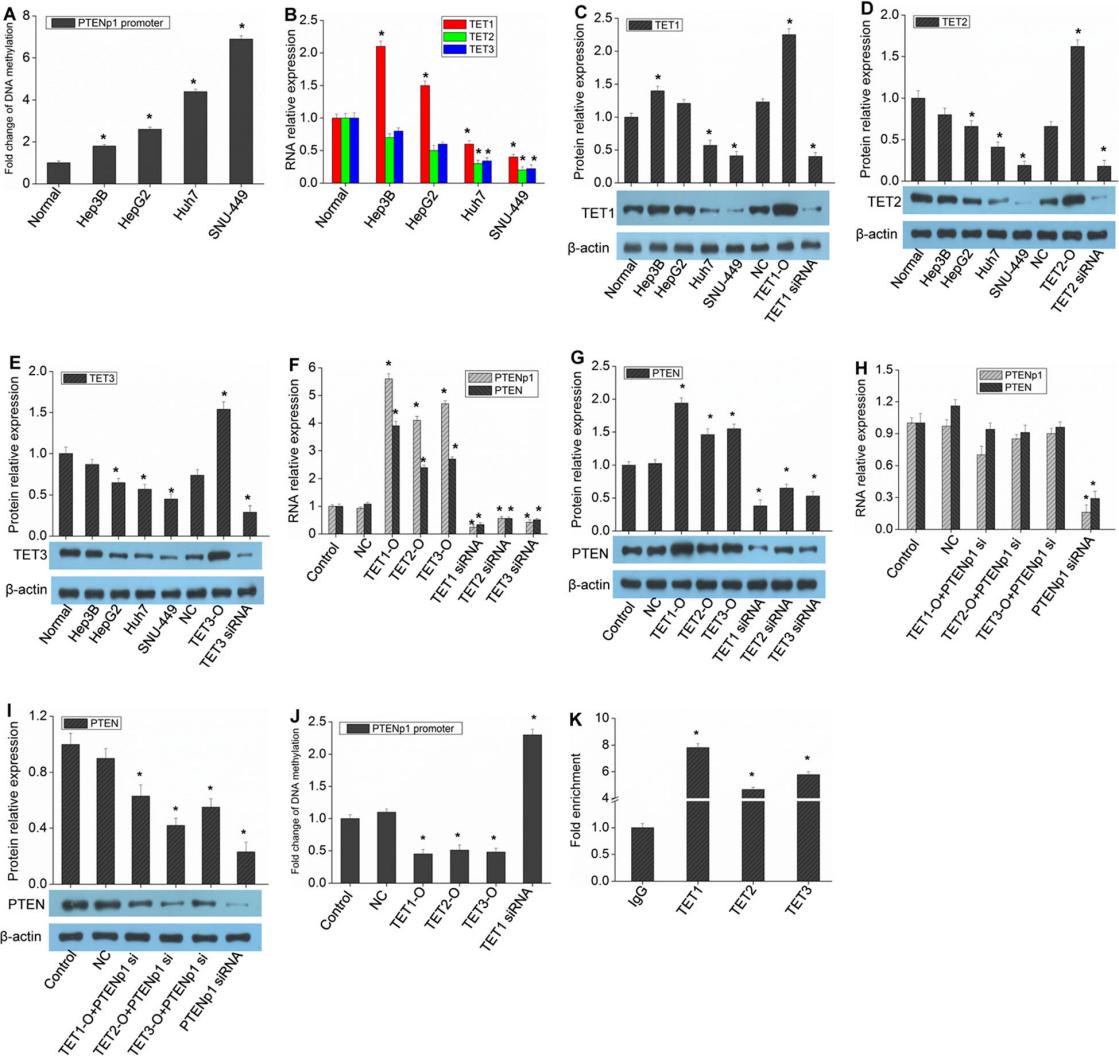
PTENp1 expression is regulated by TETs. **a** Hepatocytes and HCC cells were subjected to methylation-specific PCR assay. **b** TET1, TET2 and TET3 expression was measured by real-time qPCR analysis. **c-e** Hep3B cells were transfected with TET1-O (TET1 overexpression vector), TET2-O, TET3-O, TET1 siRNA, TET2 siRNA or TET3 siRNA and then TET1, TET2 and TET3 expression was measured by western blot analysis. **f** Hep3B cells were transfected as indicated, and then PTEN and PTENp1 expression was measured by real-time qPCR analysis. **g** PTEN expression was measured by western blot analysis. **h** Hep3B cells were transfected with TET1-O + PTENp1 siRNA, TET2-O + PTENp1 siRNA, TET3-O + PTENp1 siRNA, and PTENp1 siRNA alone and then PTEN and PTENp1 expression was measured by real-time qPCR analysis. **i** Hep3B cells were transfected as an indication, and then PTEN expression was measured by western blot analysis. **j** Hep3B cells stably infected with TET1, TET2, TET3 or TET1 shRNA lentivirus were subjected to methylation-specific PCR assay. **k** ChIP assay using anti-TET1, anti-TET2, anti-TET3 or IgG in Hep3B cells and qPCR of the PTENp1 promoter. Values correspond to the ratio between the anti-TET1, anti-TET2 or anti-TET3 antibody immunoprecipitated DNA relative to the IgG immunoprecipitated DNA. The values expressed are the mean ± SD from three samples (^*^P < 0.01, vs. normal, control or IgG)

The expression of PTENp1 is regulated not only by miR-21 but also by the degree of promoter methylation. Quantitative detection of PTENp1 promoter methylation in HCC cells showed that the methylation of PTENp1 was generally increased (Fig. 4a), which was inversely related to the expression of PTENp1. TETs are key enzymes that regulate gene demethylation. It has been reported that their expression in HCC cells is down-regulated [15]. Further detection of TET expression showed that the expression of TET family proteins was generally downregulated in HCC cells, but TET1 was expressed to a certain extent in Hep3B and HepG2 cells (Fig. 4b-e). It was further found that the upregulation of TETs could promote the expression of PTENp1 and PTEN, and the downregulation of TETs could lead to a decrease in PTENp1 and PTEN expression (Fig. 4f-g). Overexpression of TETs and then transfection of PTENp1 siRNAs dampened the promoting effect of TETs on PTEN expression (Fig. 4h-i). Therefore, TETs can regulate the expression of PTEN through PTENp1. Next, stable expression strains were constructed by infecting HCC cells with lentiviruses containing TET1, TET2 or TET3, and then the methylation of PTENp1 was quantified. It was found that the overexpression of TETs significantly reduced PTENp1 methylation (Fig. 4j). In contrast, the methylation of PTENp1 further increased after the expression of TET1 was downregulated (Fig. 4j). This indicates that TETs affect the methylation of PTENp1, which then affects the expression of PTENp1. ChIP-PCR detection showed that TETs could indeed bind to the PTENp1 promoter region (Fig. 4k). In order to verify the specificity of gene knockout, another siRNA was designed for experimental verification and the results were shown in Additional file 1: Figure S3.

### MiR-21 regulates the expression of TETs

**Fig. 5:**
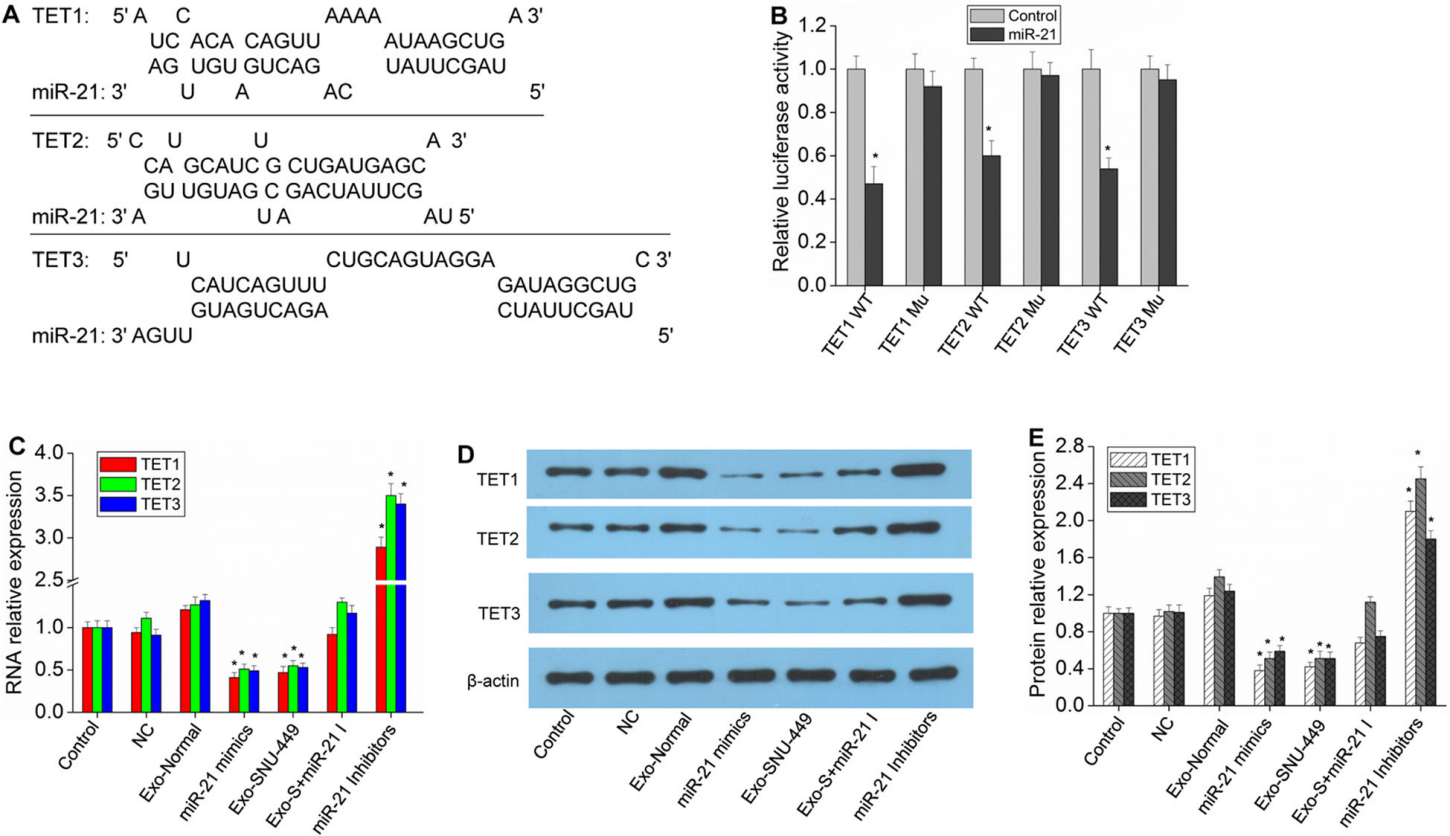
Prediction of the miR-21 target genes. **a** miR-21 target sites in the conservative sequence of TET1, TET2 and TET3. **b** The luciferase expression level of HCC cells transfected with TET1, TET2 or TET3 wild-type 3′ UTR vector (WT) or mutant 3′ UTR vector (Mu, miR-21 target sites were mutated) and miR-21 mimics. **c** Hep3B cells were transfected with miR-21 inhibitors (miR-21 I) or miR-21 mimics, then Exo-normal or Exo-SNU-449 (Exo-S) were added to the cells, and TET1, TET2 and TET3 expression was measured by real-time qPCR analysis. **d** and **e** After different treatments, TET1, TET2 and TET3 expression was measured by western blot analysis. Each bar represents the mean ± SD determined from three samples (^*^P < 0.01, vs. control)

Interestingly, further analysis revealed that miR-21 had binding sites in TET1/2/3 (Fig. 5a), and a dual-luciferase reporter gene assay confirmed the binding specificity (Fig. 5b). In HCC cells, transfection of miR-21 mimics or the addition of miR-21-containing exosomes could further reduce the expression of TETs (Fig. 5c-e). TET expression was significantly upregulated upon transfection with miR-21 inhibitors, and the inhibitory effect of exosomes on TET expression was reversed by the addition of miR-21 inhibitors (Fig. 5c-e, Additional file 1: Figure S4). Therefore, miR-21 may also affect the methylation of PTENp1 by regulating the expression of TET1/2/3, thus affecting the expression of PTENp1.

### MiR-21 regulates TETs to affect PTENp1 methylation and PTENp1 and PTEN expression

**Fig. 6:**
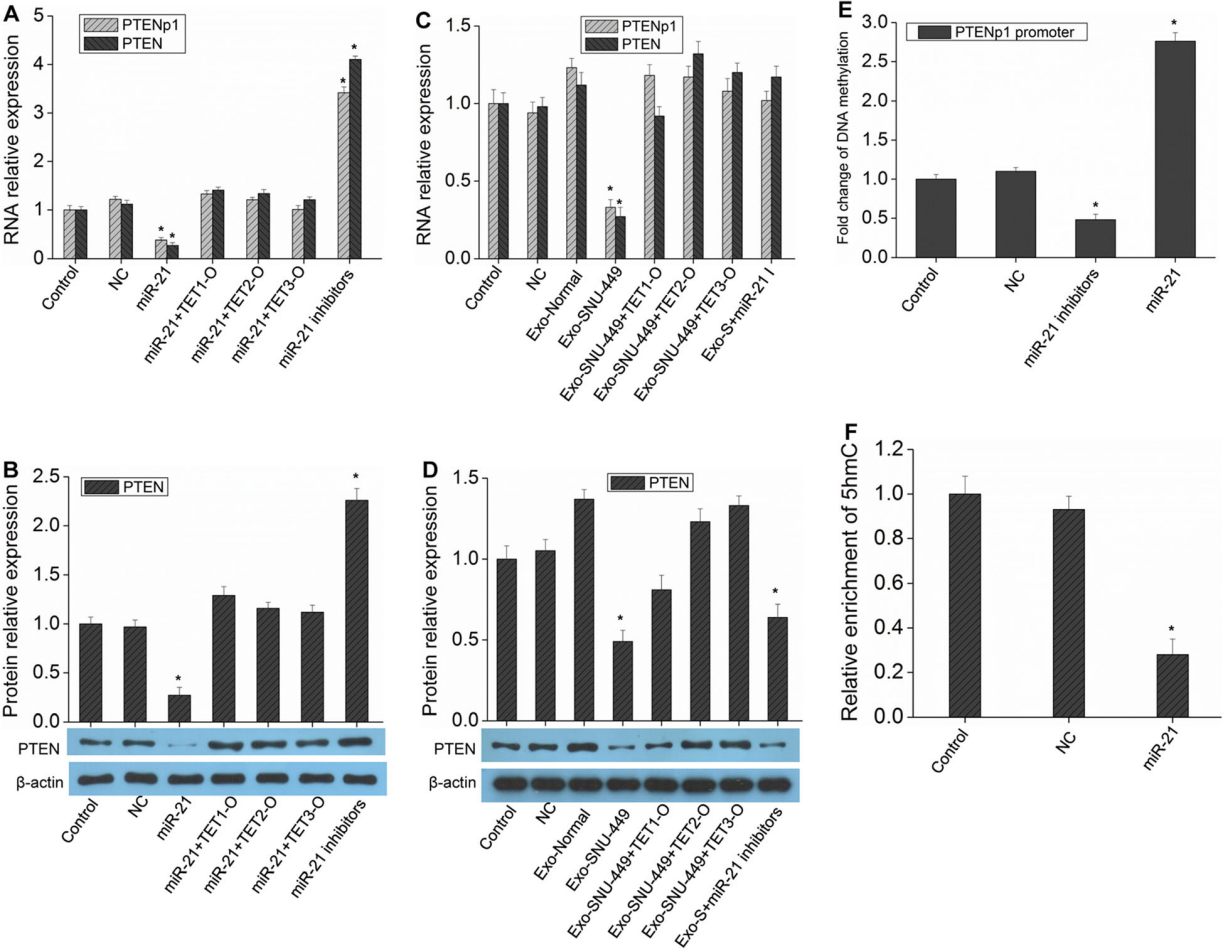
MiR-21 targets TETs to regulate the expression of PTENp1 and PTEN. **a** and **b** Hep3B cells were transfected with miR-21, miR-21 + TET1-O, miR-21 + TET2-O, miR-21 + TET3-O, or miR-21 inhibitors. Then, PTEN and PTENp1 expression was measured by real-time qPCR analysis (**a**), and western blot analysis was used to detect the expression of PTEN (**b**). **c** and **d** Hep3B cells were transfected with TET1-O, TET2-O, TET3-O or miR-21 inhibitors (miR-21 I), and then Exo-normal or Exo-SNU-449 (Exo-S) were added to the cells. PTEN and PTENp1 expression was measured by real-time qPCR analysis (**c**), and PTEN expression was measured by western blot analysis (**d**). **e** Hep3B cells stably infected with miR-21 or miR-21 inhibitor lentivirus were subjected to methylation-specific PCR assay. **f** The 5hmC level at the PTENp1 promoter upon miR-21 overexpression was analyzed using glucMS-qPCR. The values expressed are the mean ± SD from three samples (^*^P < 0.01, vs. control)

The above results suggest that miR-21 may also affect the methylation of PTENp1 and the expression of PTENp1 and PTEN by targeting TETs. The results showed that overexpression of TETs could indeed block the inhibitory effect of miR-21 on PTENp1 and PTEN expression (Fig. 6a-b). Exosomal miR-21 could inhibit the expression of PTENp1 and PTEN, and the overexpression of TETs could also dampen this effect (Fig. 6c-d). Further detection of PTENp1 methylation showed that the methylation of PTENp1 in miR-21-overexpressing HCC cells was further increased and was significantly downregulated after knockout of miR-21 expression (Fig. 6e). MiR-21 reduced the 5hmC level of the PTENp1 promoter (Fig. 6f). These results suggest that the expression of PTENp1 and PTEN can be regulated by miR-21 through targeting TETs and further affecting the methylation of PTENp1.

### MiR-21 affects HCC cell growth through the TETs/PTENp1/PTEN pathway

**Fig. 7:**
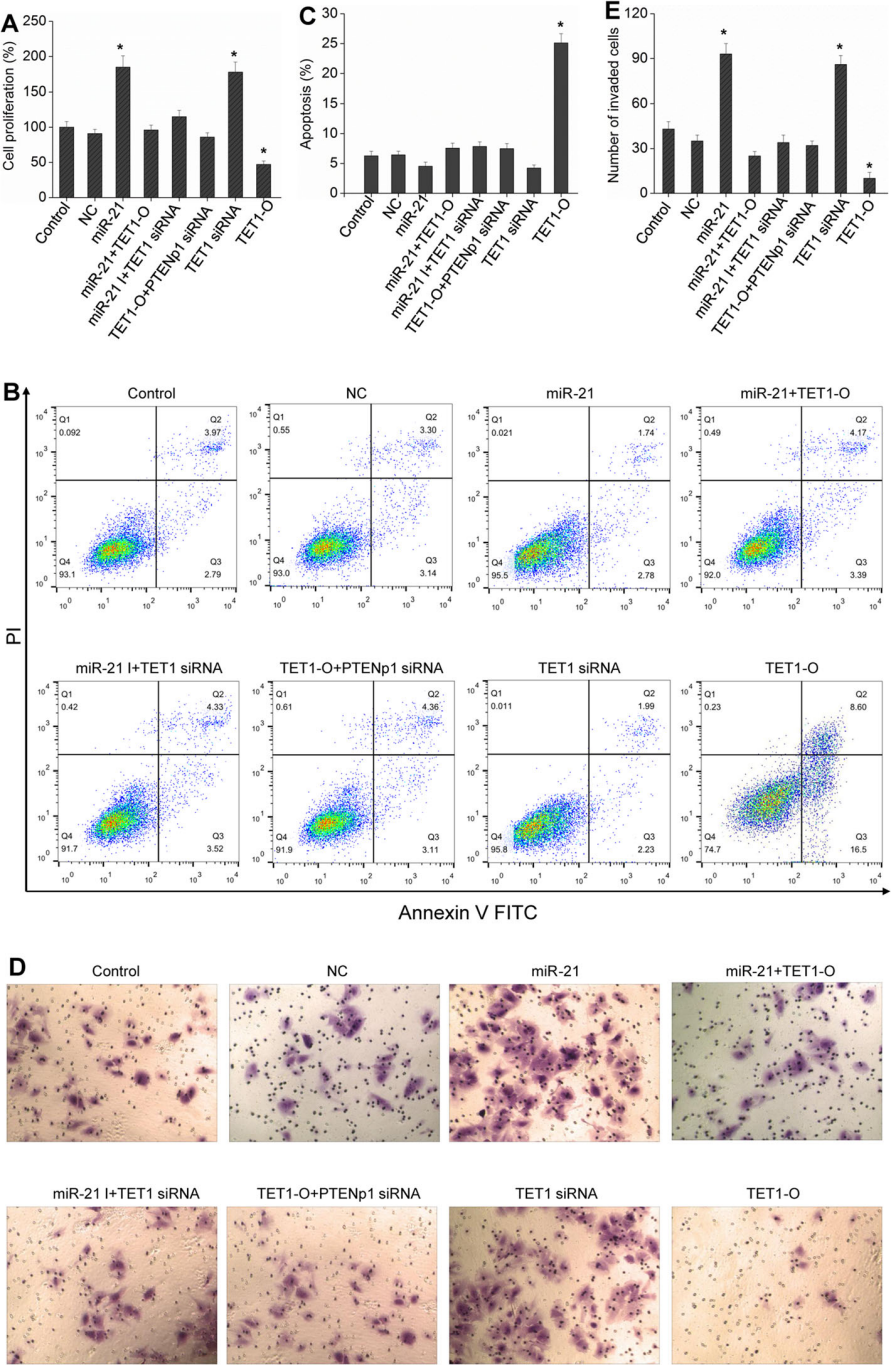
miR-21 regulates the growth of HCC cells through the TETs/PTENp1/PTEN pathway. Hep3B cells were transfected with miR-21, miR-21 + TET1-O, miR-21 I (miR-21 inhibitors) + TET1 siRNA, TET1 siRNA, TET1-O + PTENp1 siRNA, or TET1-O. Cell proliferation was examined by BrdU assay (**a**), cell apoptosis was detected by flow cytometric analysis (**b** and **c**), and cell invasion was evaluated by Transwell Matrigel invasion assay (**d** and **e**; 200 × magnification). Each bar represents the mean ± SD determined from three samples (^*^P < 0.01, vs. control)

Experiments were performed to further verify whether miR-21 can affect the growth of HCC cells by regulating TETs/PTENp1/PTEN. Both miR-21 and TET1 siRNA could promote the proliferation and downregulate the apoptosis of HCC cells, and TET1 overexpression dampened the above effect (Fig. 7a-c). Moreover, the detection of invasion showed that both miR-21 and TET1 siRNA could promote cell invasion, while TET1 overexpression inhibited the invasion of HCC cells (Fig. 7d-e). Both miR-21 and PTENp1 siRNA relieved the inhibitory effect of TET1. Therefore, miR-21 can affect the growth of HCC cells through the TETs/PTENp1/PTEN pathway.

### Effect of exosomal miR-21 on tumorigenesis in nude mice

**Fig. 8:**
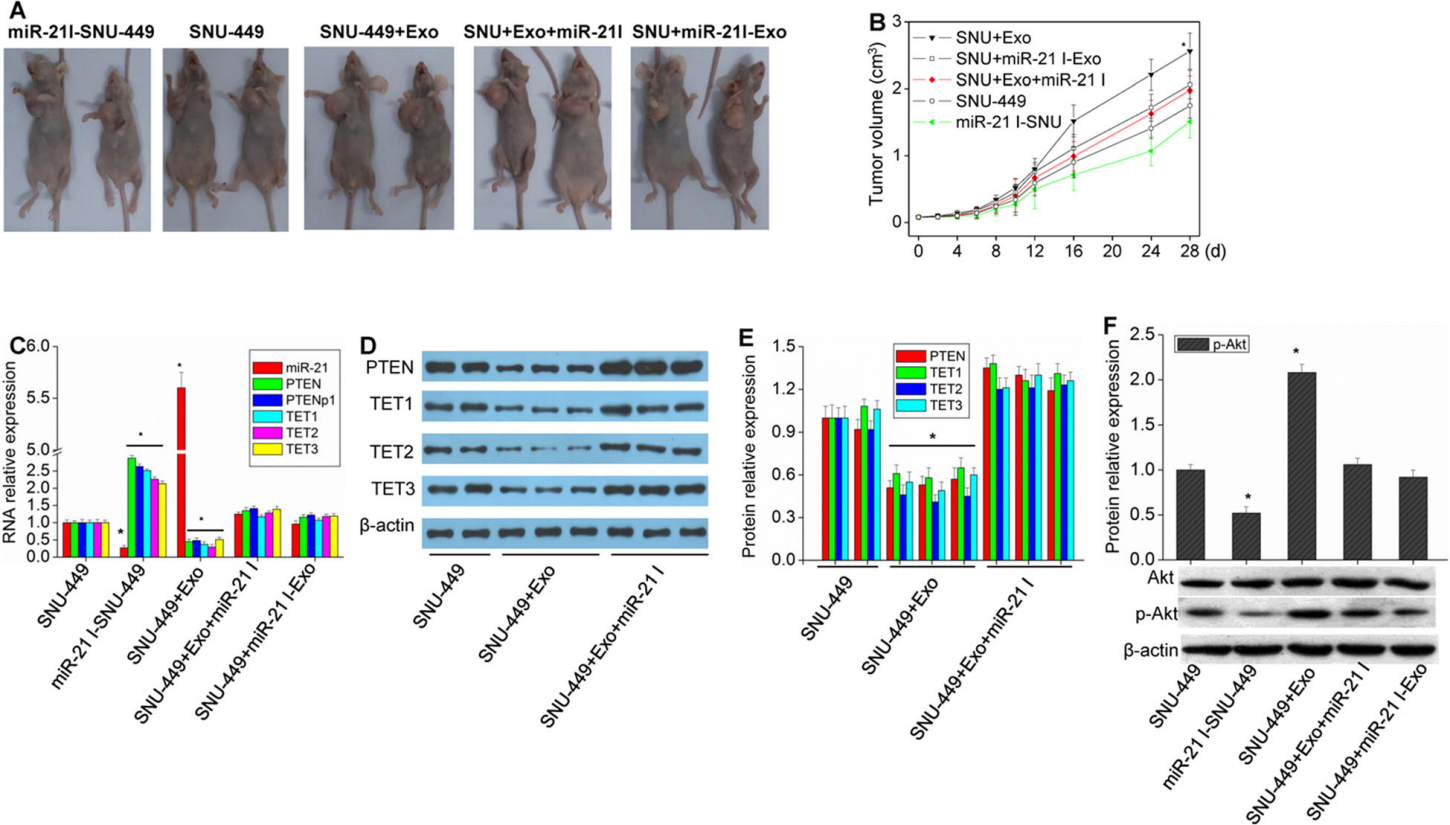
Effect of exosomal miR-21 on tumor formation in nude mice. **a** Mice were transplanted with SNU-449 cells and exosomes, as follows. miR-21I-SNU-449 (miR-21 I-SNU): Nude mice were inoculated subcutaneously with SNU-449 cells transfected with miR-21 inhibitors. SNU-449: Nude mice were inoculated subcutaneously with SNU-449 cells. SNU-449 + Exosomes (SNU-449 + Exo): Nude mice were inoculated subcutaneously with SNU-449 cells, and then SNU-449-derived exosomes were directly administered via intratumor injection. SNU-449 + Exosomes+miR-21 inhibitors (SNU + Exo + miR-21 I): Nude mice were inoculated subcutaneously with SNU-449 cells, and then exosomes transfected with miR-21 inhibitors were directly administered via intratumor injection. SNU-449 + miR-21 inhibitors-Exosomes (SNU + miR-21 I-Exo): Nude mice were inoculated subcutaneously with SNU-449 cells, and then exosomes derived from SNU-449 cells that had been transfected with miR-21 inhibitors were directly administered via intratumor injection. **b** The volumes of tumors after transplantation. ^*^P < 0.01, vs. miR-21 I-SNU. **c** miR-21, PTEN, PTENp1, TET1, TET2 and TET3 expression in tumor tissues was measured by real-time qPCR analysis. **d** and **e** PTEN, TET1, TET2 and TET3 expression in tumor tissues was measured by western blot analysis. **f** Akt and p-Akt expression in tumor tissues was measured by western blot analysis. Each bar represents the mean ± SD determined from three samples (^*^P < 0.01, vs. SNU-449)

The function of exosomal miR-21 was also verified in vivo. Injection of HCC cells and exosomes together resulted in faster growth rate of tumors and a larger tumor diameter than that of mice injected with HCC cells only (Fig. 8a-b). The results also showed that while miR-21 expression was upregulated, the expression of PTEN, PTENp1, TET1, TET2 and TET3 was downregulated (Fig. 8c-e), and the phosphorylation of Akt was increased (Fig. 8f). Next, exosomes and miR-21 inhibitors were injected with HCC cells into nude mice, and the results showed that the growth rate and size of tumors were only slightly higher than those of the group injected with HCC cells alone (Fig. 8a-b), and the expression of miR-21, PTEN, PTENp1, TET1, TET2 and TET3 was recovered (Fig. 8c-e). When SNU-449 cells were transferred with miR-21 inhibitors and then injected into nude mice, the growth rate and size of tumors were reduced (Fig. 8a-b), and the expression of PTEN, PTENp1, TET1, TET2 and TET3 was upregulated (Fig. 8c, Additional file 1: Figure S5). Therefore, exosomes can play a role in HCC through their high levels of miR-21.

## Discussion

Exosomes are nanovesicles that exist widely in the body, participate in intercellular information exchange, and affect the occurrence and development of HCC by transporting a variety of substances and regulating specific target genes [4]. Therefore, the study of the specific role of exosomes in HCC may be a new direction for HCC targeted therapy.

In this study, we found that the expression of miR-21 in exosomes was positively correlated with its expression in HCC cells and was higher than that in normal hepatocytes. Wang et al. [6] also indicated that the expression level of serum exosomal miR-21 was significantly higher in patients with HCC than those with CHB or healthy volunteers and was correlated with cirrhosis and advanced tumor stage. Therefore, exosomal miR-21 may serve as a potential biomarker for HCC diagnosis. The combination of exosomes and circulating miRNAs (miR-10b, miR-21, miR-122 and miR-200a) may serve as a promising tumor marker complementary to alpha-fetoprotein for early-stage HCC [16]. In addition, plasma exosomal miR-21 levels are a useful biomarker for the prediction of recurrence and poor prognosis in colorectal cancer patients with TNM stage II, III, or IV [17]. Hannafon et al. [18] demonstrated that miR-21 and miR-1246 were selectively enriched in human breast cancer exosomes and significantly elevated in the plasma of patients with breast cancer. Recent studies have found that compared with that in the normal population, the expression level of miR-21 in serum exosomes is significantly elevated in a variety of tumors, such as malignant gliomas and esophageal squamous cell carcinoma, suggesting that exosomal miR-21 as a marker of cancer diagnosis may become a reality [19, 20].

It was further found that exosomes derived from HCC cells could increase the expression of miR-21, inhibit the expression of PTENp1 and PTEN, promote the proliferation and migration, and inhibit the apoptosis of HCC cells. MiR-21 inhibitors partially attenuated the abovementioned effects of exosomes, suggesting that HCC cell-derived exosomes could function by transporting highly expressed miR-21. Meng et al. [9] also found that aberrant expression of miR-21 could contribute to HCC growth and spread by modulating PTEN expression and PTEN-dependent pathways involved in mediating the phenotypic characteristics of cancer cells such as cell growth, migration, and invasion. Liao et al. [21] demonstrated that delivery of exosomal miR-21 promoted cell migration and invasion by targeting PDCD4 in esophageal cancer. Au Yeung et al. [22] suggested that the malignant phenotype of metastatic ovarian cancer cells could be altered by miR-21 delivered by exosomes derived from neighboring stromal cells in the omental tumor microenvironment. Xiao et al. [23] showed that the cisplatin-resistant lung adenocarcinoma cell line A549 secreted more exosomes than the susceptible strain, and the expression of miR-21 and miR-133 increased significantly in these exosomes. Transmitting the exosomes rich in miR-21 and miR-133 of the drug-resistant strain to the susceptible strain could significantly improve the drug resistance of the latter. Therefore, exosomal miR-21 plays an important role in cell communication.

MiR-21 not only affected the expression of PTENp1 and PTEN but also significantly increased the methylation level of PTENp1. It has been found that methylation of the CpG island of PTEN is not present in many cancer cell lines but it is on the CpG island of PTENp1 [13], which is consistent with our results. For example, Wang et al. [24] found nine cases of PTEN promoter methylation in 56 specimens, but no CpG island of PTEN was found to be methylated in any of the six liver cell lines tested. Kovalenko et al. [25] also confirmed that PTEN was not methylated, while the PTENp1 pseudogene region was methylated in more than 50% of cases in endometrial cancer (11/18) and hyperplasia (5/9), but not in most normal tissues. DNA methylation modification is an important aspect of epigenetic research, and the TET protein plays an important role in the demethylation of DNA cytosine. The study also found that TET protein was down-regulated in HCC cells. Liu et al. [26] showed that the level of TET1 protein was significantly decreased in HCC tissues compared with nontumor tissues. The data by Sajadian et al. [27] clearly showed that the expression and activity of TET2 and TET3 proteins were impaired in HCC, leading to the reduction of 5hmC in HCCs. Therefore, TETs may be important tumor suppressor genes in liver cancer.

More in-depth studies have shown that miR-21 can not only directly regulate the expression of PTEN, PTENp1 and TETs but also enhance the methylation level of the PTENp1 promoter by regulating the expression of TETs, thereby inhibiting PTENp1 expression and further downregulating PTEN expression. miR-494 can trigger gene silencing of multiple invasion-suppressor miRNAs by inhibiting genomic DNA demethylation via the direct targeting of TET1, thereby leading to tumor vascular invasion [28]. Zhang et al. [29] demonstrated that miR-30a could inhibit TET1 expression through base pairing with complementary sites in the 3′ untranslated region to regulate Drp-1 promoter hydroxymethylation. Chen et al. [30] found that miR-29a silenced anti-metastatic SOCS1 through direct targeting of TET family proteins, resulting in the inhibition of SOCS1 promoter demethylation. All of the above studies suggest that miR-21 and other miRNAs can indirectly regulate the expression of some key genes by regulating the expression of TETs and influencing the methylation level of these genes. Current research shows that TETs play an important role in the occurrence and development of cancer. For example, the decreased expression of TET2 and the decrease of 5hmC content caused by the abnormal expression of miR-22 may be another important reason for hematological malignancies and solid malignancies in addition to the TET2 mutation [31]. Song et al. [32] showed that miR-22 plays an important role in the occurrence and development of solid malignant tumors such as breast cancer by inhibiting the expression of TET2.

Subsequent tumorigenesis experiments in nude mice further showed that exosomal miR-21 could promote the rapid growth of tumors, and these effects were significantly inhibited by the addition of miR-21 inhibitors. Challagundla et al. [33] reported that exosomes released by neuroblastoma (NBL) were rich in miR-21. After coculturing monocytes with these exosomes, the monocytes were injected into tumor-bearing mice, which significantly increased the resistance of tumor-bearing mice to cisplatin. Overall, exosomes with high expression of miR-21 play an important role in the development of tumors, and their uptake by target cells can affect the biological behavior of target cells by regulating the TETs/PTEN/PTENp1 signaling pathway.

## Conclusions

The results showed that exosomes with high levels of miR-21 could enhance the methylation level of the PTENp1 promoter by regulating the expression of TETs, thereby inhibiting PTENp1 expression, further down regulating PTEN expression and affecting the growth of HCC cells.

## Supplementary information

**Additional file 1: Figure S1.** Exosomes regulates HCC cell proliferation and invasion via Akt pathway. **Figure S2.** Exosomes and miR-21 regulates HCC cell proliferation and invasion. **Figure S3.** The knockdown efficiencies. **Figure S4.** Exosomal miR-21 regulates the expression of TETs. **Figure S5.** Effect of exosomal miR-21 on tumor formation in nude mice.

## Data Availability

All data in our study are available upon request.
