# Correction to: Exosomal miR-21 regulates the TETs/PTENp1/PTEN pathway to promote hepatocellular carcinoma growth

**DOI:** 10.1186/s12943-020-01177-7

**Published:** 2020-03-14

**Authors:** Liang-qi Cao, Xue-wei Yang, Yu-bin Chen, Da-wei Zhang, Xiao-Feng Jiang, Ping Xue

**Affiliations:** Department of Hepatobiliary Surgery, the Second Affiliated Hospital of Guangzhou Medical University, 250# Changgang East Road, Haizhu District, Guangzhou, 510260 People’s Republic of China

**Correction to: Mol Cancer (2019) 18:148**

**https://doi.org/10.1186/s12943-019-1075-2**

Following the publication of article [1], the authors found that the images of Transwell Matrigel invasion (Fig. 7d) are incorrect. They carelessly confused and used the images of “Control”, “miR-21 I+TET1 siRNA” and “TET1-O + PTENp1 siRNA” groups in the process of arranging diagrams. Therefore, for the scientific rigor, they want to replace these wrong images in Fig. 7 to make it clear. The authors apologize for the mistake and any inconvenience or misunderstanding that these errors may have caused, guaranteeing the image editing does not affect the integrity of the research and conclusions of the published paper.

The corrected Fig. 7 is shown on the next page:

**Fig. 7:**
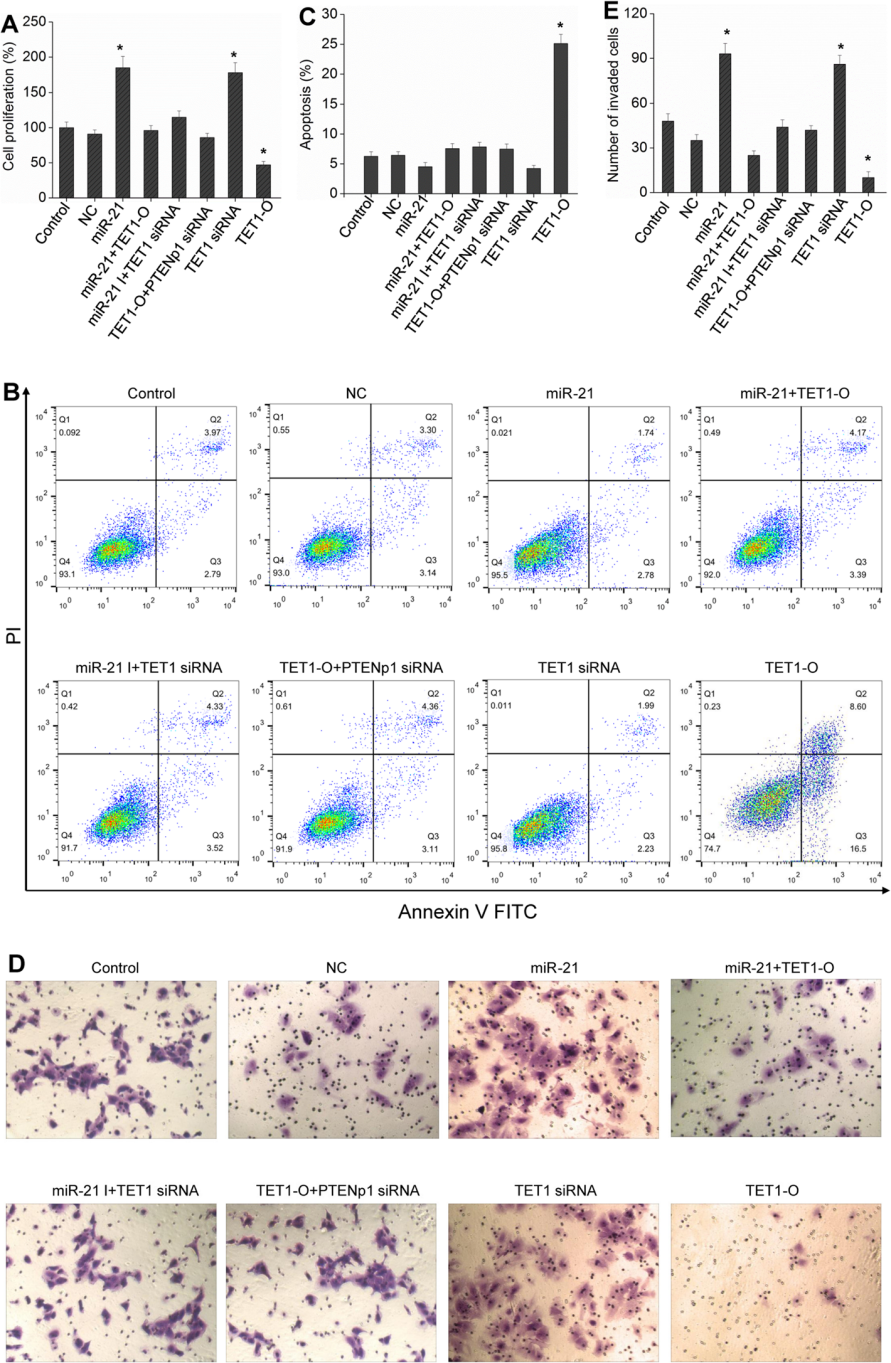
miR-21 regulates the growth of HCC cells through the TETs/PTENp1/PTEN pathway. Hep3B cells were transfected with miR-21, miR-21 + TET1-O, miR-21 I (miR-21 inhibitors) + TET1 siRNA, TET1 siRNA, TET1-O + PTENp1 siRNA, or TET1-O. Cell proliferation was examined by BrdU assay (**a**), cell apoptosis was detected by flow cytometric analysis (**b** and **c**), and cell invasion was evaluated by Transwell Matrigel invasion assay (**d** and **e**; 200 × magnification). Each bar represents the mean ± SD determined from three samples (^*^P < 0.01, vs. control)
