# Supplementary material for: Exosomal miR-21 regulates the TETs/PTENp1/PTEN pathway to promote hepatocellular carcinoma growth
Source: Mol Cancer. 2019 Oct 27;18:148. doi: 10.1186/s12943-019-1075-2 (PMC6815431; doi:10.1186/s12943-019-1075-2)
Supplement: Supplementary file 1 — Additional file 1: Figure S1. Exosomes regulates HCC cell proliferation and invasion via Akt pathway. Figure S2. Exosomes and miR-21 regulates HCC cell proliferation and invasion. Figure S3. The knockdown efficiencies. Figure S4. Exosomal miR-21 regulates the expression of TETs. Figure S5. Effect of exosomal miR-21 on tumor formation in nude mice. [file 12943_2019_1075_MOESM1_ESM.doc]

Exosomal miR-21 regulates the TETs/PTENp1/PTEN pathway to promote hepatocellular carcinoma growth

Liang-qi Cao^†^*, Xue-wei Yang^†^, Yu-bin Chen, Da-wei Zhang, Xiao-Feng Jiang, Ping Xue


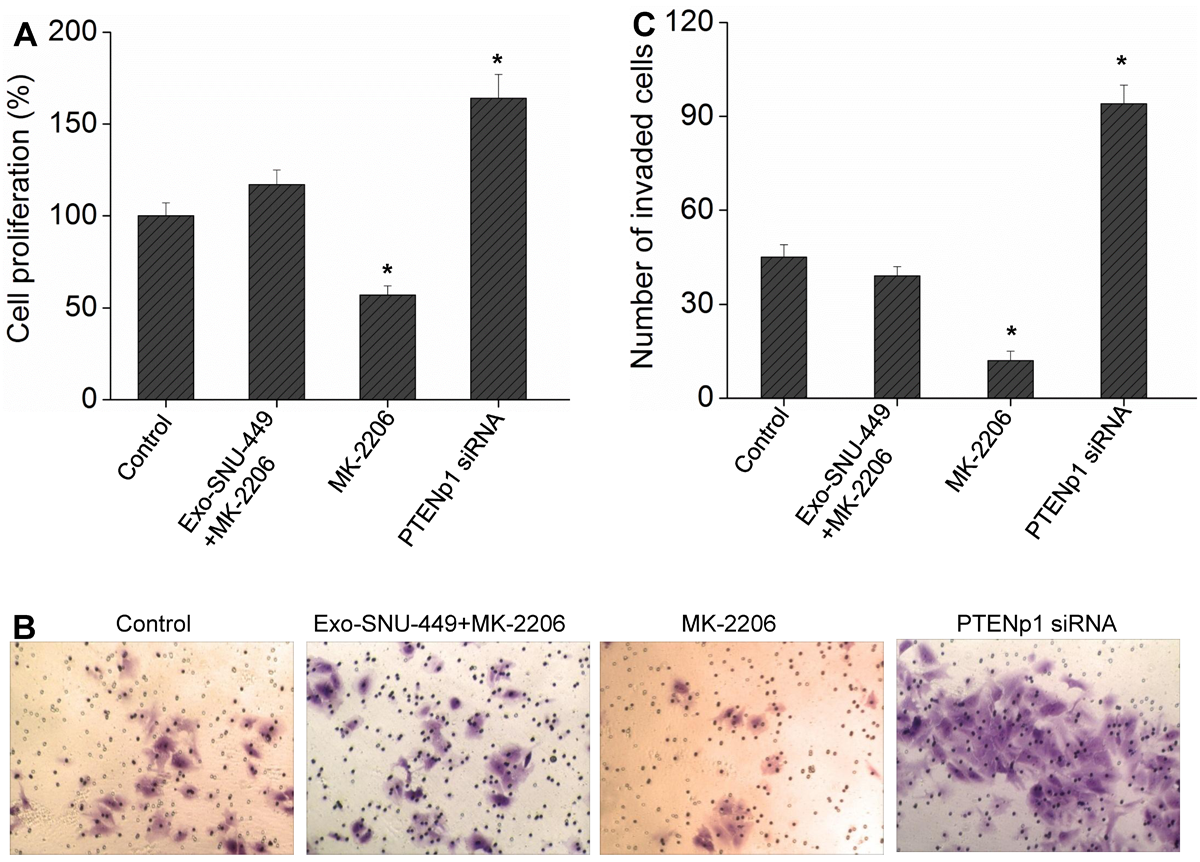


Additional file 1: **Figure S1. Exosomes regulates HCC cell proliferation and invasion via Akt pathway.** (A) Hep3B cells were cultured with exosomes obtained from SNU-449 cells (Exo-SNU-449) and MK-2206, or transfected with PTENp1 siRNA, cell proliferation was examined by BrdU assay. (B, C) Cell invasion was evaluated by Transwell Matrigel invasion assay. Each bar represents the mean ± SD determined from three samples (**P* < 0.01, vs. control).


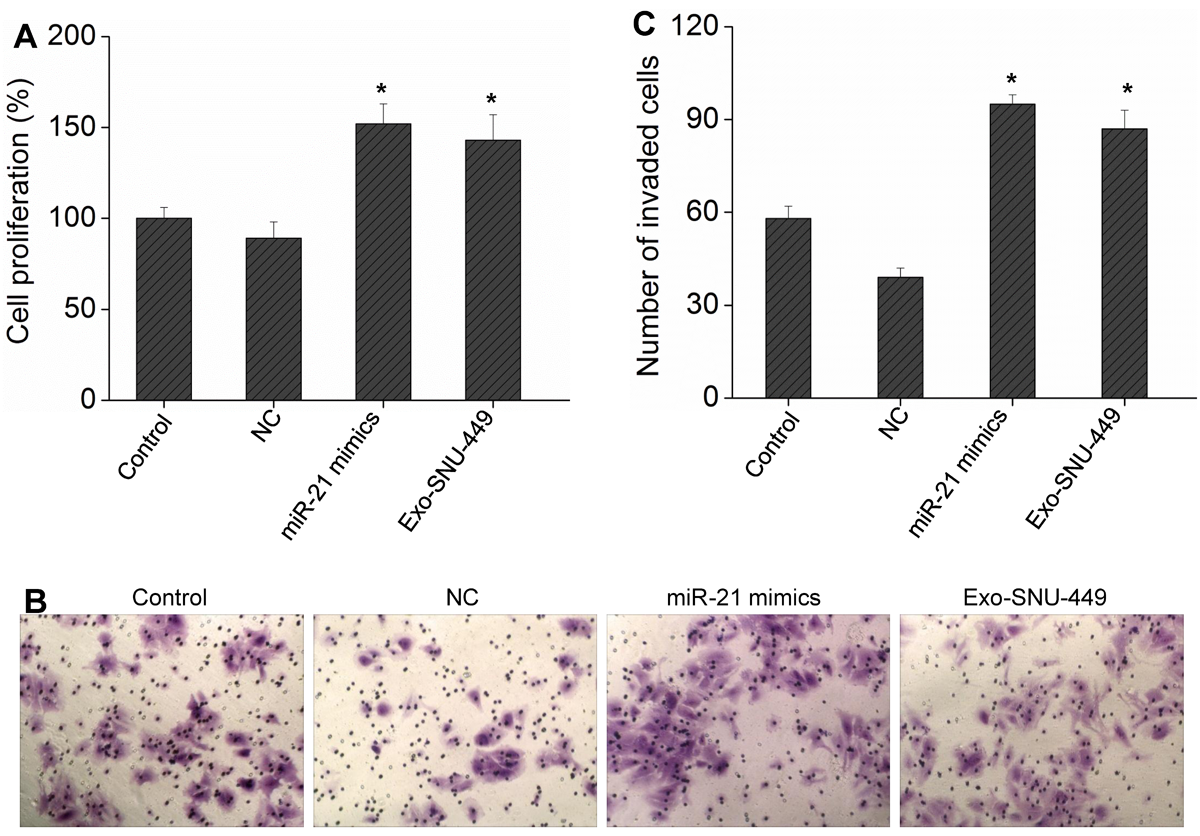


**Figure S2. Exosomes and miR-21 regulates HCC cell proliferation and invasion.** (A) Hep3B cells were infected with lentiviruses containing PTEN shRNA, then transfected with miR-21 mimics or cultured with exosomes obtained from SNU-449 cells (Exo-SNU-449), cell proliferation was examined by BrdU assay. (B, C) Cell invasion was evaluated by Transwell Matrigel invasion assay. Each bar represents the mean ± SD determined from three samples (**P* < 0.01, vs. control).


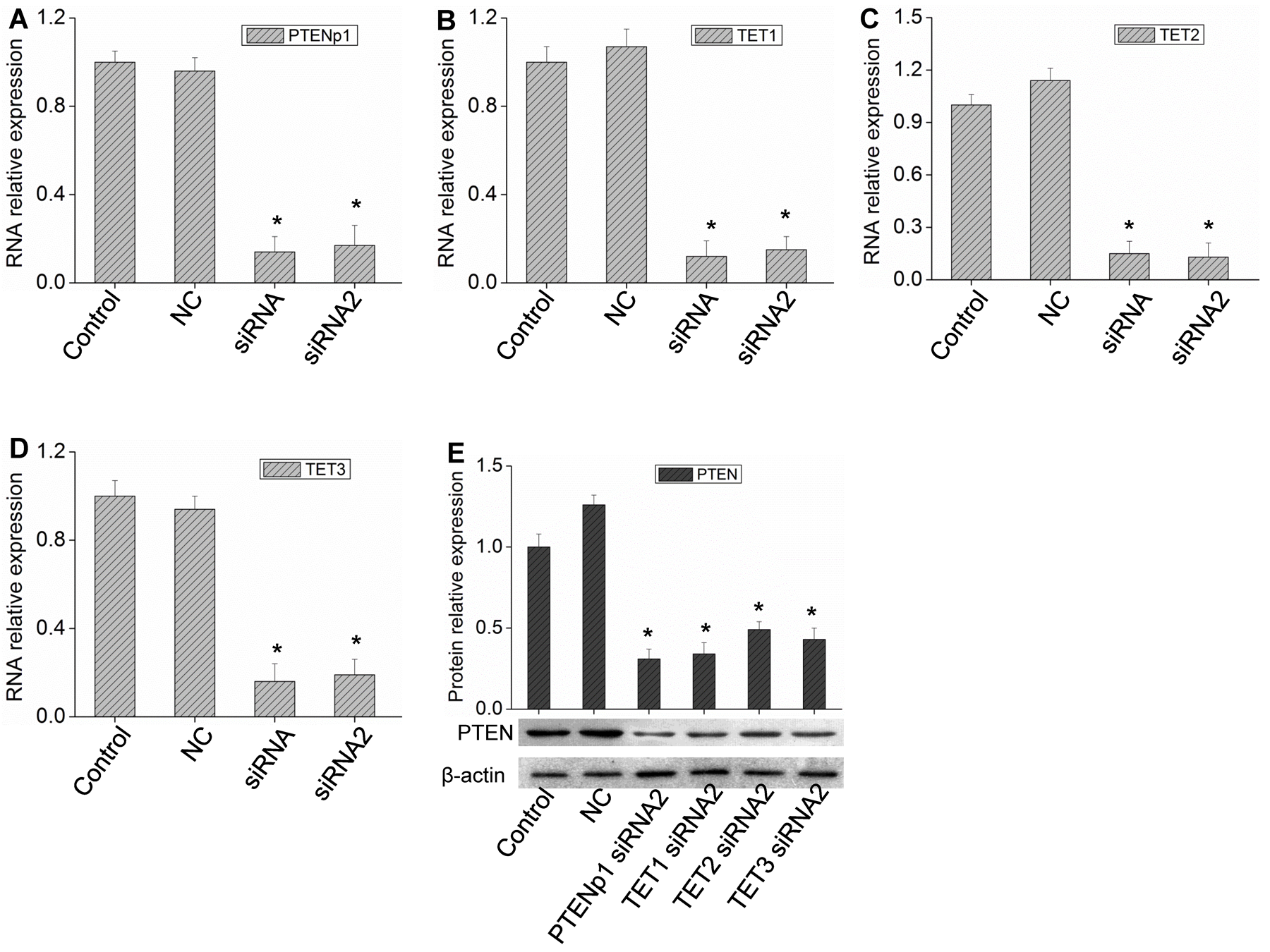


**Figure S3. The knockdown efficiencies.** (A) Hep3B cells were transfected with PTENp1 siRNA or siRNA2, and then PTENp1 expression was measured by real-time qPCR analysis. (B) Hep3B cells were transfected with TET1 siRNA or siRNA2, and then TET1 expression was measured by real-time qPCR analysis. (C) Hep3B cells were transfected with TET2 siRNA or siRNA2, and then TET2 expression was measured by real-time qPCR analysis. (D) Hep3B cells were transfected with TET3 siRNA or siRNA2, and then TET3 expression was measured by real-time qPCR analysis. (E) Hep3B cells were transfected with PTENp1, TET1, TET2 or TET3 siRNA2, and then PTEN expression was measured by western blot analysis. Each bar represents the mean ± SD determined from three samples (**P* < 0.01, vs. control).


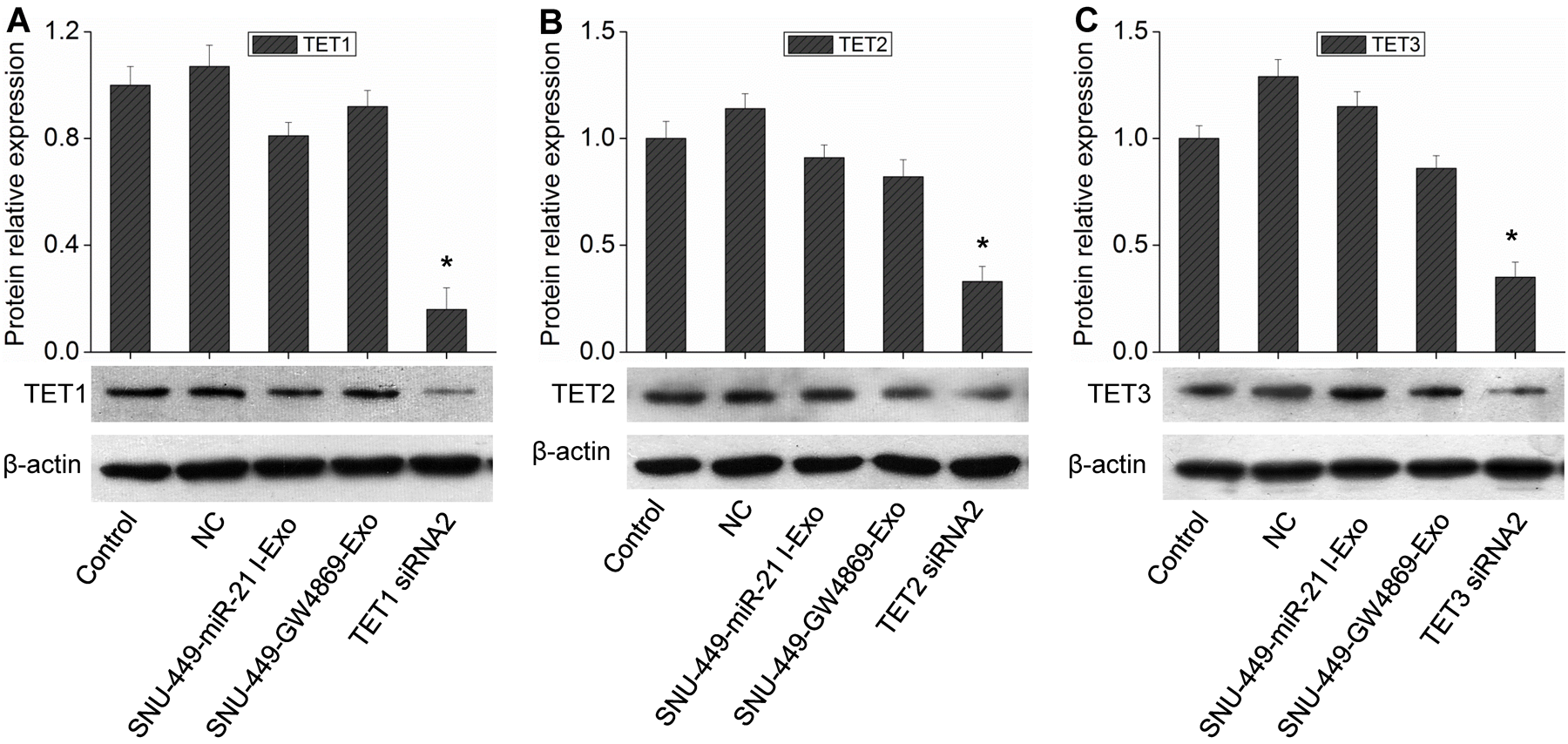


**Figure S4. Exosomal miR-21 regulates the expression of TETs.** Hep3B cells were cultured with exosomes obtained from SNU-449 cells transfected with miR-21 inhibitors (SNU-449-miR-21 I-Exo) or SNU-449 cells added with GW4869 (SNU-449-GW4869-Exo), or transfected with TET1, TET2 and TET3 siRNA2. TET1 (A), TET2 (B) and TET3 (C) expression was measured by western blot analysis. Each bar represents the mean ± SD determined from three samples (**P* < 0.01, vs. control).


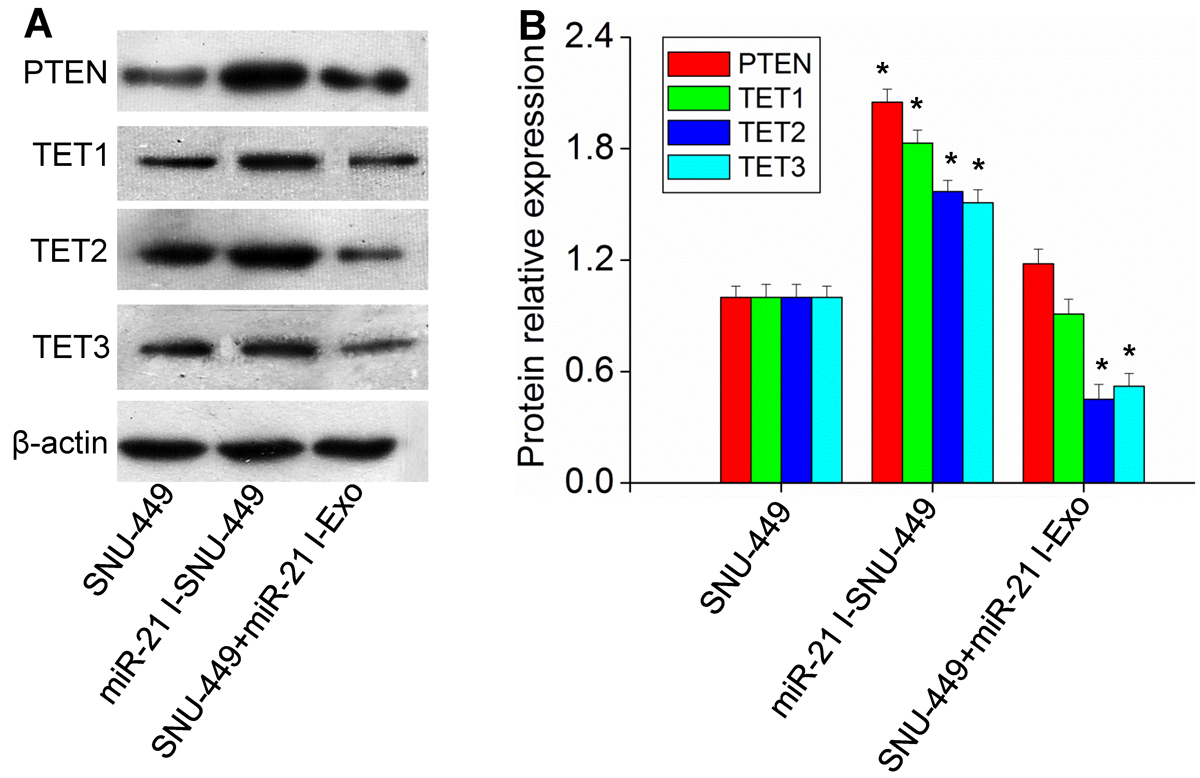


**Figure S5. Effect of exosomal miR-21 on tumor formation in nude mice.** (A) Mice were transplanted with SNU-449 cells and exosomes as follows. SNU-449: Nude mice were inoculated subcutaneously with SNU-449 cells. miR-21 I-SNU-449: Nude mice were inoculated subcutaneously with SNU-449 cells transfected with miR-21 inhibitors. SNU-449+miR-21 inhibitors-Exosomes (SNU+miR-21 I-Exo): Nude mice were inoculated subcutaneously with SNU-449 cells, and then exosomes derived from SNU-449 cells that had been transfected with miR-21 inhibitors were directly administered via intratumor injection. PTEN, TET1, TET2 and TET3 expression was measured by western blot analysis. (B) Quantitative comparison of protein expression levels. Each bar represents the mean ± SD determined from three samples (**P* < 0.01, vs. SNU-449).
